# Innate Immune Cell Infiltration Induced by Polatuzumab Vedotin Contributes to the Antitumor Effect in Mouse Models

**DOI:** 10.1002/jha2.70219

**Published:** 2026-01-19

**Authors:** Mayu Tomita, Natsumi Kawasaki, Keigo Yorozu, Sei Shu, Chie Kato, Mitsue Kurasawa, Bansho Masutani, Nicolas Sax, Yoriko Yamashita‐Kashima, Shigeki Yoshiura

**Affiliations:** ^1^ Product Research Department Chugai Pharmaceutical Co. Ltd Yokohama Japan; ^2^ Biological Technology Department Chugai Pharmaceutical Co. Ltd Yokohama Japan

**Keywords:** DLBCL, immunogenic cell death, innate immune cells, polatuzumab vedotin, tumor microenvironment

## Abstract

**Introduction:**

Polatuzumab vedotin (Pola) is an antibody‐drug conjugate approved for the treatment of diffuse large B‐cell lymphoma (DLBCL). Several reports suggest that the tumor microenvironment influences the outcome of DLBCL treatments; with Pola, however, the link between tumor microenvironment and treatment outcome remains unclear.

**Objectives and Methods:**

We analyzed the relationship between the antitumor effect of Pola and immune status, focusing on innate immune cells in the tumor microenvironment by utilizing a xenograft mouse model and a syngeneic mouse model.

**Results:**

In the DB (DLBCL cell line) xenograft model, Pola induced infiltration of macrophages and natural killer cells, which contributed to the antitumor effect of Pola. Moreover, Pola induced the release of several damage‐associated molecular patterns from DB cells and enhanced the migration of immune cells under ex vivo co‐culture conditions. A syngeneic mouse model also confirmed the involvement of innate immune cells in the Pola effect.

**Conclusion:**

This study demonstrates that Pola treatment alters MΦ and NK cell infiltration in tumors, highlighting these innate immune cells' essential contribution to Pola's antitumor activity.

**Trial Registration:**

The authors have confirmed clinical trial registration is not needed for this submission

## 1 Introduction

Diffuse large B‐cell lymphoma (DLBCL) represents almost 30% of all cases of non‐Hodgkin's lymphoma, and most patients present with advanced‐stage disease [1]. Currently, the treatment landscape of DLBCL is being transformed by novel drug therapies such as chimeric antigen receptor (CAR) T‐cell therapy, antibody‐drug conjugates (ADCs), and bispecific antibodies [2, 3, 4]. Polatuzumab vedotin (Pola) is an ADC comprising an anti‐CD79b antibody linked with monomethyl auristatin E (MMAE), a potent microtubule polymerization inhibitor [5, 6]. The global Phase 3 POLARIX study showed that previously untreated DLBCL patients receiving Pola, rituximab (Rit), cyclophosphamide, doxorubicin, and prednisone (Pola‐R‐CHP) had significantly higher progression‐free survival rates than those who received Rit, cyclophosphamide, doxorubicin, vincristine, and prednisone (R‐CHOP) [7]. Based on that study, Pola is approved in many countries for the first‐line treatment of DLBCL as a component of the Pola‐R‐CHP regimen.

Previous studies suggest vedotin‐based ADCs may modify tumor microenvironments through immunogenic cell death (ICD) [8, 9]. Another study has suggested that treatment with vedotin‐based ADCs results in robust immunomodulatory changes in vivo, accompanied by increased infiltration of innate immune cells that are distinct from those produced by other clinical ADC payloads [10]. However, few studies have focused on the interaction between the anti‐tumor effect of Pola and the tumor immune microenvironment in DLBCL.

In this study, we analyzed the relationship between the antitumor effect of Pola and immune status, focusing on innate immune cells, including natural killer (NK) cells and macrophages (MΦs) that play important roles in the cancer immune cycle [11], by utilizing xenograft and syngeneic mouse models.

## 2 Methods

### 2.1 Cell Lines

The DB (human) and L1210 (mouse) cell lines were purchased from the American Type Culture Collection. DB cells were cultured in RPMI‐1640 ATCC modification (Thermo Fisher Scientific) with 10% fetal bovine serum (Nichirei Biosciences or Sigma, or Corning). L1210 cells were cultured in Dulbecco's Modified Eagle's Medium High Glucose (Sigma) supplemented with 10% horse serum (Thermo Fisher Scientific) and 1 mM sodium pyruvate (Thermo Fisher Scientific). All cells were cultured at 37°C under 5% CO_2_.

### 2.2 Animals and In Vivo Experiments

All animal experiments were reviewed and approved by the Institutional Animal Care and Use Committee at Chugai Pharmaceutical Co. Ltd., an institute accredited by AAALAC International. Practices at our institute conform to those stipulated in the *Guide for the Care and Use of Laboratory Animals* published by the Institute for Laboratory Animal Research.

CB‐17/Icr‐scid/scidJcl mice (scid; female, 5‐week‐old), NOD/Shi‐scid, IL‐2RγKO mice (NOG; female, 5‐week‐old), and DBA/2 j mice (female, 7–8‐week‐old) were obtained from CLEA Japan. Scid and NOG mice were inoculated subcutaneously with 5 × 10^6^ DB or #5‐1 cells. DBA/2 j mice were inoculated subcutaneously with 5 × 10^6^ L1210‐hCD79b‐9 cells.

After tumor engraftment, mice were randomized into groups, and Pola (Chugai Pharmaceutical Co. Ltd.) and/or human IgG (huIgG, MP Biomedicals) were administered intravenously on Day 1. To evaluate the antitumor activity of the test agents, tumor volume was assessed as described previously [12]. Tumor regression was defined as tumor shrinkage at the endpoint relative to the pre‐treatment volume (Day 0 or 1).

To evaluate the contribution of MΦs to the antitumor effect of Pola, 1.5 mg/mouse clodronate liposomes (Liposoma) or 1.5 mg/mouse control liposomes (Liposoma) were intravenously injected on Days 0 and 2 (in the DB xenograft model) or on Days 0, 2, 4, and 7 (in the L1210‐hCD79b‐9 syngeneic mouse model). To evaluate the contribution of NK cells to the antitumor effect of Pola, 100 µg/mouse anti‐asialo GM1 (Fujifilm Wako Chemicals) or control rabbit serum (Fujifilm Wako Chemicals) was intraperitoneally injected on Day 0 (in the DB xenograft model) or on Days 0, 4, and 7 (in the L1210‐hCD79b‐9 syngeneic mouse model).

### 2.3 Establishment of New Cell Lines

Details of methods are supplied in the Supporting Information. Briefly, the Pola‐resistant #5‐1 cell line was developed from DB tumors via continuous Pola administration to a scid mouse. The L1210‐hCD79b‐9 (Pola sensitive) cell line was established by overexpressing a human‐mouse chimeric CD79b gene sequence, including the Pola‐recognizing site (indicated in [13] as a human CD79b peptide recognized by SN8) of human CD79b in L1210 cells.

### 2.4 Cell Viability Assay

Cells were incubated with Pola in varying concentrations. After 3 or 4 days, CellTiter‐Glo 3 D Cell Viability Assay (Promega) was added to each well, and luminescence was measured using a Varioskan LUX Multimode Microplate Reader (Thermo Fisher Scientific). For each drug concentration test, cell viability was calculated as a percentage of this control value using the formula: (signal at test concentration / signal at 0 µg/mL) × 100%.

### 2.5 Immunohistochemistry

Sections of tumor paraffin blocks (harvested on Day 4) were stained with NCR1, CD68, and granzyme B (GzmB) antibodies, and apoptotic cells were assessed by TUNEL (TdT‐mediated dUTP nick end labeling) assay, as described in the Supporting Information.

### 2.6 Ex Vivo Co‐Culture Experiments With MΦs Isolated From Tumors

MΦs were isolated from tumor xenograft tissues in the control (ctrl) IgG or Pola group on Day 4 according to a method for collecting adherent cells as described elsewhere [14] after the isolation of CD45^+^ cells using CD45 (TIL) MicroBeads, mouse (Miltenyi Biotec). Isolated MΦs were co‐cultured with target DB cells at an effector/target ratio of 10:1. One day after co‐culture, the apoptosis level of DB cells was determined by using RealTime‐Glo Annexin V Apoptosis and Necrosis Assay (Promega) with a Varioskan LUX Multimode Microplate Reader.

### 2.7 Antibody‐Dependent Killing Assay

Antibody‐dependent killing of target cells, such as through antibody‐dependent cell‐mediated cytotoxicity (ADCC) and antibody‐dependent cellular phagocytosis (ADCP), is a rapid process, and the ADCC and ADCP activity of antibodies can be determined within 24 h by co‐culturing target cells with effector cells and antibodies [15, 16]. Therefore, antibody‐dependent killing was assessed using a co‐culture system of labeled DB cells and CD45^+^ effector cells for 16 h as described in the Supporting Information.

### 2.8 In Vitro DAMPs Analysis

DB cells were incubated with Pola or MMAE (Selleck) or anti‐human CD79b antibody (SN8, BD Bioscience) in varying concentrations. After 2 or 3 days, extracellular ATP (eATP) was assessed by using RealTime‐Glo Extracellular ATP Assay (Promega) and High Mobility Group Box 1 (HMGB1) was assessed by using Lumit HMGB1 Human/Mouse Immunoassay (Promega). The total fluorescence intensity was determined with a Varioskan LUX Multimode Microplate Reader.

To detect surface‐exposed calreticulin, DB cells were stained with PE Anti‐Calreticulin (cat. no. ab209577; Abcam) 3 days after Pola treatment in varying concentrations. Stained cells were analyzed with a BD LSRFortessa X‐20 cell analyzer and FlowJo v10 software.

### 2.9 Migration Assay of CD45^+^ Immune Cells

Immune cell migration ability was determined by a transwell migration assay system (CytoSelect 24‐well Cell Migration Assay [8 µm], colorimetric; Cell Biolabs) (Figure 3D, right panel). Chambers with a porous membrane were suspended over larger wells that contained 0 or 0.1 µg/mL Pola with or without DB cells. Then, CD45^+^ cells isolated from the DB tumors (by using CD45 [TIL] MicroBeads, mouse) were placed inside the chamber and allowed to migrate through the pores for 3 days. Migrated cells attached to the membrane were then stained with stain solution, and the number of these cells per field was counted under an inverted microscope (DMi1; Leica).

### 2.10 scRNA‐Sequencing

Single‐cell RNA sequencing (scRNA‐seq) by Chromium Fixed RNA Profiling was performed on CD45^+^ immune cells from DB and #5‐1 tumors treated with 2 mg/kg ctrl IgG or 2 mg/kg Pola. Details of methods are provided in the Supporting Information.

### 2.11 Statistical Analysis

For data other than scRNA‐seq data, Student's *t*‐test was used for two‐group comparisons, Dunnett's test was used for multiple comparisons with the control group, and Tukey's honestly significant difference (HSD) test was used for multiple comparisons. Statistical analyses using Student's *t*‐test, Dunnett's test, and Tukey's HSD test were performed with statistical software packages JMP v15.0 or v17.2 (SAS Institute).

## 3 Results

### 3.1 Pola‐Induced Infiltration of Innate Immune Cells Contributes to the Antitumor Effect of Pola in a Human DLBCL Xenograft Model

**FIGURE 1:**
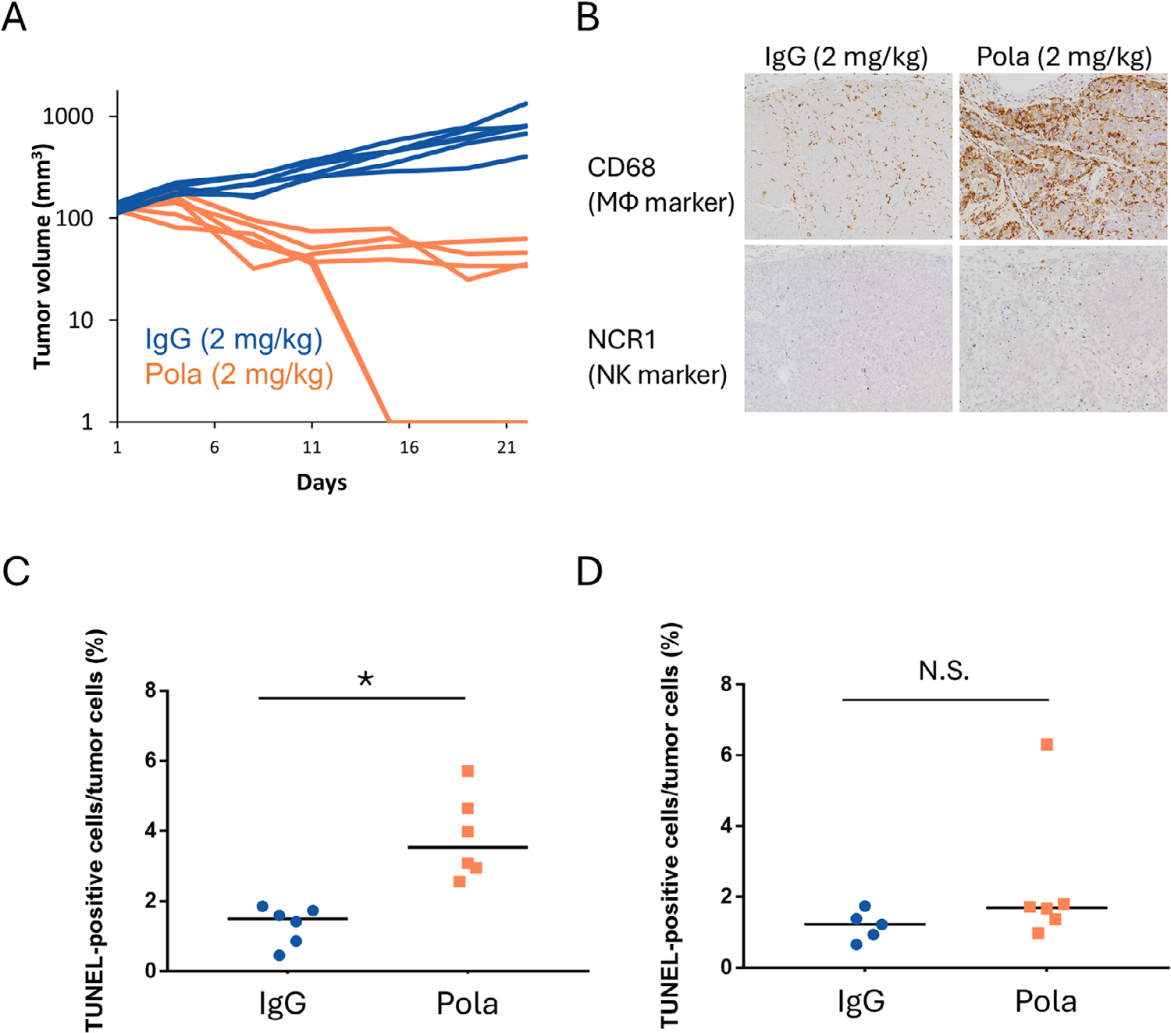
Pola increased infiltration of MΦs and NK cells into tumors, and innate immune cells contributed to the antitumor effect of Pola in the DB xenograft model in scid mice. (A) In vivo DB tumor growth curves from Day 1 to Day 22 after administration of 2 mg/kg ctrl IgG or 2 mg/kg Pola (*n* = 6). (B) Representative images of CD68 or NCR1 expression on Day 4 in the 2 mg/kg ctrl IgG or 2 mg/kg Pola treatment group as assessed by IHC. Original magnification ×10. (C, D) Percentage of TUNEL‐positive cells among tumor cells in the 2 mg/kg ctrl IgG or 2 mg/kg Pola treatment group in mice administered either (C) control liposomes plus normal rabbit serum or (D) clodronate liposomes plus anti‐asialo GM1 (*n* = 5–6). *p < 0.05 by Student's *t*‐test; N.S.; not significant. In dot plots, horizontal bars represent median values. In vivo experiments (A–D) were conducted in scid mice.

First, we analyzed the relationship between the antitumor effect of Pola and innate immune cell status in scid mice xenografted with DB (a generally and widely used human DLBCL cell line for investigating drug efficacy), which lack adaptive immune cells but maintain innate immune cells. In this model, regression of DB tumors was observed on Day 22 in the 2 mg/kg Pola treatment group (six out of six mice) but not in the ctrl IgG group (Figure 1A). The levels of MΦs and NK cell infiltration were higher in the Pola treatment group than in the ctrl IgG group on Day 4 (Figures 1B and 4C,D).

To investigate the role that this Pola‐induced infiltration of innate immune cells plays in the antitumor effect of Pola, we detected DNA fragmentation by TUNEL assays to evaluate apoptotic cells under conditions where mice had been injected with clodronate liposomes, which deplete MΦ levels [17], and anti‐asialo GM1 antibody, which depletes NK cells [18]. The infiltration levels of MΦ and NK cells following administration of clodronate liposomes plus anti‐asialo GM1 are shown in Figure S1. Under non‐depleted conditions, the TUNEL assay on Day 4 showed a significant increase in the ratio of apoptotic cells in the 2 mg/kg Pola treatment group as compared to the ratio in the ctrl IgG group (Figure 1C). In contrast, under depleted MΦs and NK cell conditions, no significant increase was observed in the ratio of apoptotic cells in the 2 mg/kg Pola treatment group (Figure 1D). These data suggest that Pola‐induced infiltration of innate immune cells contributed to the anti‐tumor effect of Pola in DB tumors.

### 3.2 Innate Immune Cells Infiltrating DB Tumors Have the Potential to Induce Apoptotic Cell Death

Next, we assessed the characteristics of MΦs and NK cells infiltrating DB tumors to reveal how these innate immune cells contributed to the antitumor effect.

**FIGURE 2:**
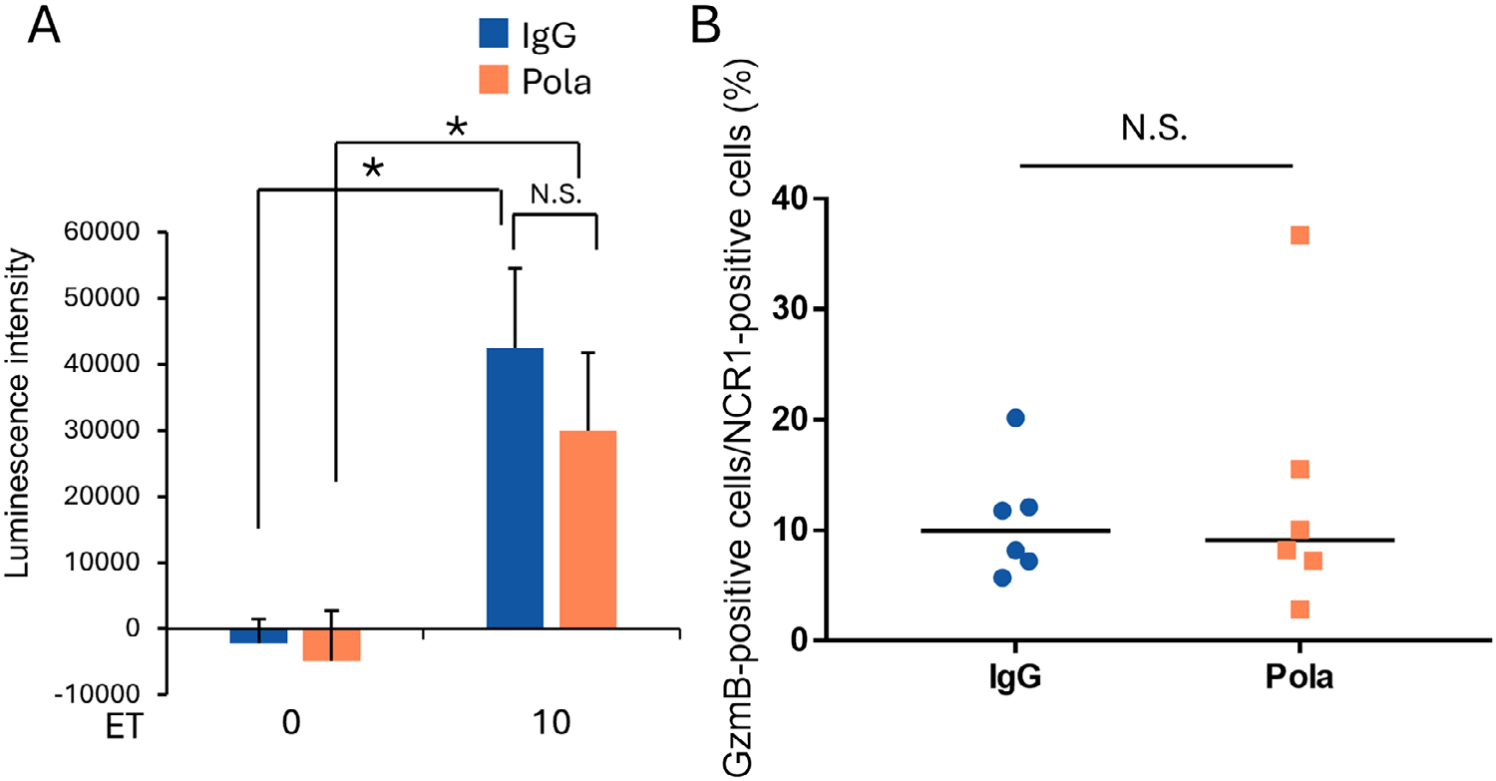
Innate immune cells infiltrating DB tumors have the potential to induce apoptotic cell death. (A) Levels of apoptosis in DB cells co‐cultured ex vivo with MΦs isolated from tumors from the 2 mg/kg ctrl IgG‐treated or 2 mg/kg Pola‐treated DB xenograft models (*n* = 6). ET; effector/target ratio. The data represent the mean + SD; *p < 0.05 by Tukey's HSD test. (B) Percentage of GzmB‐positive cells among NCR1‐positive cells in the 2 mg/kg ctrl IgG or 2 mg/kg Pola treatment groups as assessed by IHC (*n* = 6). N.S.; not significant by Student's *t*‐test. In dot plots, horizontal bars represent median values. Ex vivo and in vivo experiments were conducted using scid mice.

The ability of MΦs to induce apoptosis in tumor cells was analyzed by ex vivo co‐culture experiments using DB cells and MΦs isolated from tumors. MΦs isolated from DB tumors were capable of inducing apoptosis in DB cells, and there was no difference in the level of induced apoptosis between Pola‐treated and ctrl IgG‐treated groups (Figure 2A). The activation status of tumor‐infiltrating NK cells was analyzed by Immunohistochemistry (IHC). We found that the subset of NK cells in DB tumors was GzmB‐positive, indicating that they had been activated and possessed cytotoxic potential (Figure 2B). However, there was no difference between the Pola‐treated and ctrl IgG‐treated groups in the proportion of GzmB‐positive cells among intratumoral NK cells (Figure 2B). Moreover, scRNA‐seq analysis comparing the status of MΦs and NK cells in DB tumors from the Pola‐treated group with those from the IgG‐treated group revealed no differentially expressed genes (DEGs) in either the MΦs or the NK cells (Figure S2).

The antibody‐dependent killing assay using Pola and CD45^+^ immune cells isolated from DB tumors revealed that DB cells would not be killed by mechanisms dependent on the Fc domain of the anti‐CD79b antibody component of Pola in this model (Figure S3), as has been indicated previously [6]. These results suggest that MΦs and NK cells infiltrating DB tumors have the potential to induce apoptotic cell death in tumor cells, regardless of Pola treatment status.

### 3.3 Pola Induces the Release of DAMPs From DB Cells and Enhances Migration Activity of Immune Cells In Vitro

**FIGURE 3:**
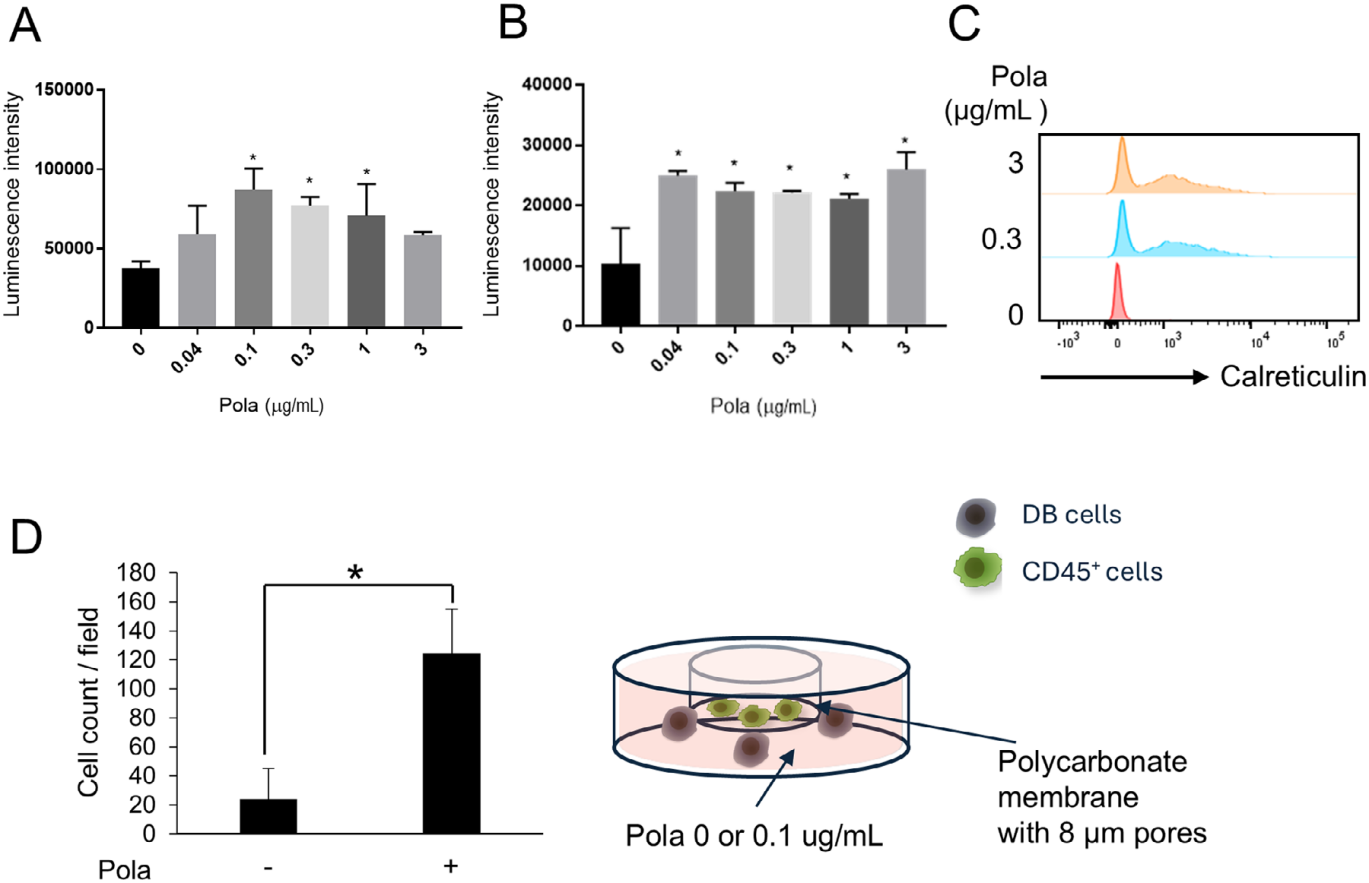
Pola induced the release of DAMPs from DB cells in vitro. Levels of (A) eATP (3 replicates) and (B) HMGB1 (2 replicates) released from DB cells 3 days after Pola treatment in vitro. The data represent the mean + SD; *p < 0.05 by Dunnet's test compared to Pola 0 µg/mL. (C) Levels of surface‐exposed calreticulin on DB cells 3 days after Pola treatment in vitro. (D, left panel) Migration ability of CD45^+^ immune cells seeded onto a porous membrane cell culture insert in the presence of 0 or 0.1 µg/mL Pola with DB cells (3 replicates) below the membrane. The data represent the mean + SD; *p < 0.05 by Student's *t*‐test. (D, right panel) Schematic diagram of the migration assay.

Recent studies have suggested that some vedotin‐based ADCs induce ICD of tumor cells and increase innate immune cell infiltration [8, 9, 10]. ICD results in cell death accompanied by the passive release of numerous damage‐associated molecular patterns (DAMPs), including surface‐exposed calreticulin as well as eATP and HMGB1 [19]. eATP is known as a “find‐me” signal to recruit MΦs [20], and HMGB1 is known to induce migration of MΦs [21]. Therefore, we assessed the involvement of DAMPs in the change of levels of immune cell infiltration induced in DB tumors by Pola. Pola‐induced DB cells were found to release eATP, HMGB1, and express calreticulin on the cell surface (Figure 3A–C). Importantly, treatment with the anti‐human CD79b antibody SN8, the parental antibody for Pola, which shares the same antigen‐binding domain as Pola, did not induce the release of DAMPs, whereas treatment with the payload component (MMAE) resulted in significant DAMPs release, suggesting that the cytotoxic payload is responsible for triggering ICD (Figure S4).

Using transwell co‐culture assays, we analyzed whether Pola‐treated DB cells enhanced the migration ability of CD45^+^ cells, including MΦs and NK cells, and found that Pola‐treated DB cells enhanced the migration ability of CD45^+^ cells (Figure 3D). Therefore, the infiltration of MΦs and NK cells into DB tumors could be caused by the ability of DB cells to recruit immune cells via DAMPs released upon Pola‐induced ICD.

### 3.4 Levels of Pola‐Induced Innate Immune Cell Infiltration Correlate With the Antitumor Effect of Pola in Pola‐Sensitive and Low‐Pola‐Sensitivity Tumor Xenograft Models

**FIGURE 4:**
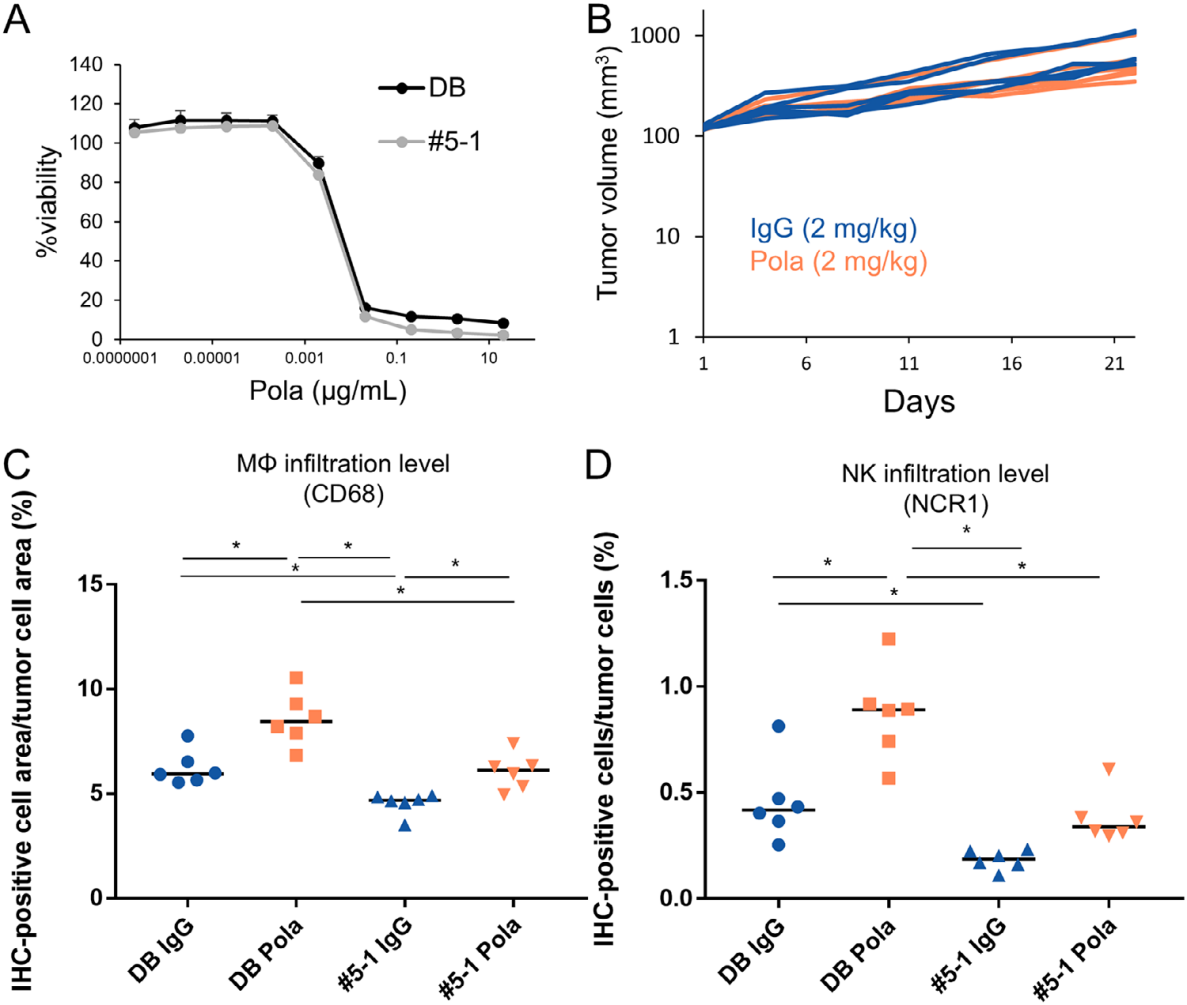
Pola induced a low level of infiltration of innate immune cells in #5‐1 tumors as compared to that in DB tumors. (A) In vitro sensitivity to Pola at the indicated concentrations in DB and #5‐1 cells. The data represent the mean + SD (3 replicates). (B) #5‐1 tumor growth curves from Day 1 to Day 22 after administration of 2 mg/kg ctrl IgG or 2 mg/kg Pola (*n* = 6). (C) Percentage of the area of CD68‐positive staining cells to the total area of tumor cells and (D) percentage of NCR1‐positive cells among tumor cells on Day 4 after 2 mg/kg ctrl IgG or 2 mg/kg Pola administration in DB and #5‐1 models (*n* = 6). *p < 0.05 by Tukey's HSD test. In dot plots, horizontal bars represent median values. In vivo experiments (B–D) were conducted in scid mice.

To further investigate the influence of the tumor microenvironment on Pola efficacy, we generated an in vivo specific Pola low‐sensitivity model (#5‐1 cell line) and compared DB tumors with #5‐1 tumors. There is no difference in Pola sensitivity between DB and #5‐1 cells in vitro (Figure 4A).

In contrast to DB tumors (Figure 1A), no regression of #5‐1 tumors in scid mice was observed in the 2 mg/kg Pola treatment group by Day 22 (Figure 4B). Gene Ontology enrichment analysis based on scRNA‐seq data showed that movement‐related pathways of MΦs and NK cells were significantly suppressed in #5‐1 tumors as compared to those in DB tumors (Table 1). In scid mice, #5‐1 tumors showed significantly lower intratumoral infiltration of MΦs and NK cells compared to DB tumors, in both ctrl IgG‐treated groups and Pola‐treated groups (Figure 4C,D). These results suggest that the baseline immunosuppressive environment of #5‐1 tumors could be the cause of the low level of immune cell infiltration induced by Pola compared to that in DB tumors.

**TABLE 1:** Enrichment of movement‐related pathways in MΦs and NK cells, comparing 2 mg/kg ctrl IgG‐treated #5‐1 tumors with 2 mg/kg ctrl IgG‐treated DB tumors (*n* = 4). Only statistically significant pathways (*p < 0.1) are shown in this list.

Term	Overlap	**Adjusted *p*‐value**	**Odds ratio**	**Combined score**	**Genes**	**Activation_type**	**Cell_type**
GOBP_LEUKOCYTE_MIGRATION	12/269	3.76406E‐06	9.351363847	154.4411801	*Csf1r*;*Vcam1*;*Cd74*;*Csf3r*;*Tlr2*;*Abl1*;*Fpr2*;*Cd24a*;*C5ar1*;*Pla2g7*;*Aif1*;*Lyn*	Down	NK
GOBP_POSITIVE_REGULATION_OF_LEUKOCYTE_MIGRATION	8/118	1.21353E‐05	13.96936692	201.344029	*Csf1r*;*Cd74*;*Tlr2*;*Abl1*;*Fpr2*;*C5ar1*;*Pla2g7*;*Aif1*	Down	NK
GOBP_REGULATION_OF_LEUKOCYTE_MIGRATION	9/168	1.21353E‐05	10.95871106	154.4556122	*Csf1r*;*Cd74*;*Tlr2*;*Abl1*;*Fpr2*;*C5ar1*;*Pla2g7*;*Aif1*;*Lyn*	Down	NK
GOBP_MYELOID_LEUKOCYTE_MIGRATION	9/173	1.21353E‐05	10.62041677	147.0704747	*Csf1r*;*Cd74*;*Csf3r*;*Tlr2*;*Fpr2*;*C5ar1*;*Pla2g7*;*Aif1*;*Lyn*	Down	NK
GOBP_REGULATION_OF_LEUKOCYTE_CHEMOTAXIS	7/89	1.21353E‐05	16.26640316	223.4240659	*Csf1r*;*Cd74*;*Fpr2*;*C5ar1*;*Pla2g7*;*Aif1*;*Lyn*	Down	NK
GOBP_REGULATION_OF_CHEMOTAXIS	8/144	2.0427E‐05	11.27987941	145.515284	*Csf1r*;*Fn1*;*Cd74*;*Fpr2*;*C5ar1*;*Pla2g7*;*Aif1*;*Lyn*	Down	NK
GOBP_POSITIVE_REGULATION_OF_CHEMOTAXIS	7/101	2.0427E‐05	14.18426501	182.6663043	*Csf1r*;*Fn1*;*Cd74*;*Fpr2*;*C5ar1*;*Pla2g7*;*Aif1*	Down	NK
GOBP_CELL_CHEMOTAXIS	9/205	2.7371E‐05	8.863008046	110.2575604	*Csf1r*;*Vcam1*;*Cd74*;*Csf3r*;*Fpr2*;*C5ar1*;*Pla2g7*;*Aif1*;*Lyn*	Down	NK
GOBP_POSITIVE_REGULATION_OF_LEUKOCYTE_CHEMOTAXIS	6/71	2.7371E‐05	17.48176421	215.6227928	*Csf1r*;*Cd74*;*Fpr2*;*C5ar1*;*Pla2g7*;*Aif1*	Down	NK
GOBP_LEUKOCYTE_MIGRATION	21/269	3.13049E‐05	4.131962036	60.41023657	*Pde4b*;*S100a9*;*Cxcr2*;*Tnfrsf18*;*Cd81*;*Pecam1*;*Vcam1*;*Cd74*;*Gpr18*;*Ccl5*;*Itgal*;*S100a8*;*Asb2*;*Pde4d*;*Sell*;*Trem3*;*Tbx21*;*Icam1*;*Cxcl13*;*Thy1*;*Itgb3*	Down	Macrophage0
GOBP_LEUKOCYTE_CHEMOTAXIS	8/163	3.32456E‐05	9.883248442	118.3688706	*Csf1r*;*Cd74*;*Csf3r*;*Fpr2*;*C5ar1*;*Pla2g7*;*Aif1*;*Lyn*	Down	NK
GOBP_CELL_MOTILITY	19/1020	3.32456E‐05	4.009485771	47.78041448	*Csf1r*;*Trib1*;*Abl1*;*Pak1*;*Zeb2*;*Fn1*;*Cd24a*;*P2ry2*;*Pla2g7*;*Aif1*;*Vcam1*;*Cd74*;*Fpr2*;*C5ar1*;*Lyn*;*Csf3r*;*Tlr2*;*Il1rn*;*Pltp*	Down	NK
GOBP_REGULATION_OF_MONOCYTE_CHEMOTAXIS	4/21	3.32456E‐05	44.00543093	521.5537682	*Pla2g7*;*Fpr2*;*Aif1*;*Lyn*	Down	NK
GOBP_CELL_MOTILITY	45/1020	0.000125945	2.3335278	29.250811	*Pde4b*;*Pex13*;*Arsb*;*S100a9*;*Cxcr2*;*Plekhg5*;*Fscn1*;*F2r*;*Cxcr6*;*Tnfrsf18*;*Egr3*;*Cd81*;*Sema4a*;*Smurf2*;*Muc2*;*Adgrg1*;*Epha2*;*Lcn2*;*Syne2*;*Pecam1*;*Arf4*;*Vcam1*;*Ets1*;*Cd74*;*Cadm4*;*Gpr18*;*Ccl5*;*Itgal*;*S100a8*;*Eomes*;*Pde4d*;*Tbxa2r*;*Wnt11*;*Asb2*;*Sell*;*Prcp*;*Trem3*;*Tbx21*;*Icam1*;*Pdgfa*;*Cxcl13*;*Thy1*;*Itgb3*;*Celsr1*;*Rapgef2*	Down	Macrophage0
GOBP_NEUTROPHIL_CHEMOTAXIS	10/77	0.000150605	7.184965831	85.86558938	*Pde4b*;*Cd74*;*Trem3*;*S100a9*;*Ccl5*;*Cxcr2*;*S100a8*;*Cxcl13*;*Pde4d*;*Sell*	Down	Macrophage0
GOBP_NEUTROPHIL_MIGRATION	11/98	0.000155005	6.086315789	70.47125191	*Pde4b*;*Cd74*;*Trem3*;*S100a9*;*Ccl5*;*Cxcr2*;*S100a8*;*Cxcl13*;*Pde4d*;*Pecam1*;*Sell*	Down	Macrophage0
GOBP_CELL_CHEMOTAXIS	16/205	0.000155005	4.085007384	46.61446891	*Vcam1*;*Pde4b*;*Cd74*;*Cxcr6*;*Trem3*;*Gpr18*;*S100a9*;*Ccl5*;*Egr3*;*Cxcr2*;*S100a8*;*Plekhg5*;*Cxcl13*;*Epha2*;*Pde4d*;*Sell*	Down	Macrophage0
GOBP_MONOCYTE_CHEMOTAXIS	4/40	0.000429398	21.05977584	194.0348465	*Pla2g7*;*Fpr2*;*Aif1*;*Lyn*	Down	NK
GOBP_LYMPHOCYTE_MIGRATION	6/87	0.065018477	3.612865593	15.9566894	*Tbx21*;*Icam1*;*Ccl5*;*Itgal*;*Cxcl13*;*Itgb3*	Down	Macrophage0
GOBP_REGULATION_OF_LEUKOCYTE_CHEMOTAXIS	6/89	0.067027885	3.525633297	15.20282856	*Cd74*;*Gpr18*;*Cxcr2*;*Ccl5*;*Cxcl13*;*Sell*	Down	Macrophage0
GOBP_POSITIVE_REGULATION_OF_MONOCYTE_CHEMOTAXIS	3/16	0.000465745	43.6630533	395.5071975	*Pla2g7*;*Fpr2*;*Aif1*	Down	NK
GOBP_POSITIVE_REGULATION_OF_LEUKOCYTE_CHEMOTAXIS	5/71	0.090573995	3.735435471	14.67857003	*Cd74*;*Cxcr2*;*Ccl5*;*Cxcl13*;*Sell*	Down	Macrophage0
GOBP_REGULATION_OF_NEUTROPHIL_CHEMOTAXIS	3/27	0.090573995	6.421633554	24.8998498	*Sell*;*Cd74*;*Cxcr2*	Down	Macrophage0
GOBP_TROPHOBLAST_CELL_MIGRATION	2/11	0.095326924	11.79467901	44.41551631	*Smurf2*;*Itgb3*	Down	Macrophage0
GOBP_POSITIVE_REGULATION_OF_MONONUCLEAR_CELL_MIGRATION	4/57	0.001487797	14.34424963	112.283346	*Pla2g7*;*Abl1*;*Fpr2*;*Aif1*	Down	NK
GOBP_POSITIVE_REGULATION_OF_LEUKOCYTE_MIGRATION	10/118	0.003055087	4.451833346	36.71731081	*Cd74*;*Tnfrsf18*;*Ccl5*;*Icam1*;*Cxcr2*;*Cxcl13*;*Thy1*;*Itgb3*;*Pecam1*;*Sell*	Down	Macrophage0
GOBP_REGULATION_OF_LEUKOCYTE_MIGRATION	12/168	0.003241652	3.690463075	29.65013007	*Cd74*;*Tnfrsf18*;*Gpr18*;*Ccl5*;*Icam1*;*Cd81*;*Cxcr2*;*Cxcl13*;*Thy1*;*Itgb3*;*Pecam1*;*Sell*	Down	Macrophage0
GOBP_POSITIVE_REGULATION_OF_NEUTROPHIL_MIGRATION	3/33	0.003685878	19.29454885	132.2836079	*Cd74*;*C5ar1*;*Tlr2*	Down	NK
GOBP_MYELOID_LEUKOCYTE_MIGRATION	12/173	0.003707073	3.574427956	27.76103161	*Pde4b*;*Cd74*;*Trem3*;*S100a9*;*Ccl5*;*Cxcr2*;*Cd81*;*S100a8*;*Cxcl13*;*Pde4d*;*Pecam1*;*Sell*	Down	Macrophage0
GOBP_REGULATION_OF_NEUTROPHIL_MIGRATION	3/41	0.006545421	15.27346637	95.01818859	*Cd74*;*C5ar1*;*Tlr2*	Down	NK
GOBP_LEUKOCYTE_CHEMOTAXIS	11/163	0.007154496	3.469715272	24.25776814	*Pde4b*;*Cd74*;*Trem3*;*Gpr18*;*S100a9*;*Ccl5*;*Cxcr2*;*S100a8*;*Cxcl13*;*Pde4d*;*Sell*	Down	Macrophage0
GOBP_NEUTROPHIL_MIGRATION	4/98	0.009400214	8.088547816	46.92977653	*Cd74*;*Csf3r*;*C5ar1*;*Tlr2*	Down	NK
GOBP_POSITIVE_REGULATION_OF_MACROPHAGE_CHEMOTAXIS	2/18	0.01567792	25.10181818	131.4435287	*Csf1r*;*C5ar1*	Down	NK
GOBP_POSITIVE_REGULATION_OF_MACROPHAGE_MIGRATION	2/23	0.022998414	19.25488372	91.52152895	*Csf1r*;*C5ar1*	Down	NK
GOBP_REGULATION_OF_MACROPHAGE_CHEMOTAXIS	2/23	0.022998414	19.25488372	91.52152895	*Csf1r*;*C5ar1*	Down	NK
GOBP_NEUTROPHIL_CHEMOTAXIS	3/77	0.028537944	7.865498991	35.01207453	*Cd74*;*Csf3r*;*C5ar1*	Down	NK
GOBP_REGULATION_OF_NEUTROPHIL_CHEMOTAXIS	2/27	0.028537944	16.22823529	72.07751599	*Cd74*;*C5ar1*	Down	NK
GOBP_POSITIVE_REGULATION_OF_ACTIN_FILAMENT_BASED_MOVEMENT	1/2	0.028537944	163.2992126	719.1401021	*Zeb2*	Down	NK
GOBP_MACROPHAGE_CHEMOTAXIS	2/32	0.036558539	13.56131148	55.80908706	*Csf1r*;*C5ar1*	Down	NK
GOBP_NEGATIVE_REGULATION_OF_SMOOTH_MUSCLE_CELL_CHEMOTAXIS	1/3	0.036580966	97.97007874	392.0149663	*Aif1*	Down	NK
GOBP_NEURAL_CREST_CELL_MIGRATION_INVOLVED_IN_AUTONOMIC_NERVOUS_SYSTEM_DEVELOPMENT	1/3	0.036580966	97.97007874	392.0149663	*Fn1*	Down	NK
GOBP_SUBSTRATE_DEPENDENT_CELL_MIGRATION_CELL_ATTACHMENT_TO_SUBSTRATE	1/3	0.036580966	97.97007874	392.0149663	*Fn1*	Down	NK
WP_REGULATION_OF_ACTIN_CYTOSKELETON	3/94	0.038094672	6.393575903	25.05633625	*Pak1*;*Cd14*;*Fn1*	Down	NK
GOBP_POSITIVE_REGULATION_OF_LYMPHOCYTE_MIGRATION	2/36	0.038094672	11.98434783	46.6411062	*Abl1*;*Aif1*	Down	NK
GOBP_REGULATION_OF_MACROPHAGE_MIGRATION	2/37	0.038820296	11.6456338	44.72129008	*Csf1r*;*C5ar1*	Down	NK
GOBP_T_CELL_MIGRATION	5/55	0.045484938	4.926701802	24.47978017	*Icam1*;*Ccl5*;*Itgal*;*Cxcl13*;*Itgb3*	Down	Macrophage0
GOBP_ENDOTHELIAL_CELL_CHEMOTAXIS	3/19	0.045484938	9.542644993	46.31964228	*Plekhg5*;*Cxcl13*;*Egr3*	Down	Macrophage0
GOBP_NEGATIVE_CHEMOTAXIS	3/19	0.045484938	9.542644993	46.31964228	*Pdgfa*;*Sema4a*;*Itgb3*	Down	Macrophage0
GOBP_REGULATION_OF_T_CELL_MIGRATION	2/41	0.04558514	10.46227848	38.16418724	*Abl1*;*Aif1*	Down	NK
GOBP_REGULATION_OF_SMOOTH_MUSCLE_CELL_CHEMOTAXIS	1/5	0.049907152	54.41732283	190.2746387	*Aif1*	Down	NK
GOBP_SMOOTH_MUSCLE_CELL_CHEMOTAXIS	1/5	0.049907152	54.41732283	190.2746387	*Aif1*	Down	NK
GOBP_MACROPHAGE_MIGRATION	2/49	0.057937799	8.693473684	28.84832654	*Csf1r*;*C5ar1*	Down	NK
GOBP_REGULATION_OF_LYMPHOCYTE_MIGRATION	2/52	0.062776962	8.174653465	26.24063657	*Abl1*;*Aif1*	Down	NK
GOBP_T_CELL_MIGRATION	2/55	0.067612592	7.714018692	23.97821576	*Abl1*;*Aif1*	Down	NK

**FIGURE 5:**
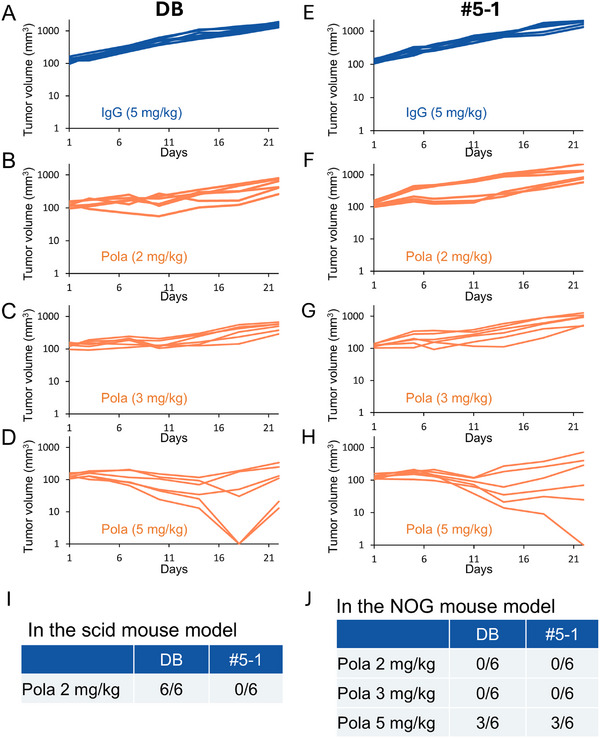
The difference between DB and #5‐1 tumor regression by Pola treatment observed in the scid mouse model was diminished in the NOG mouse model. (A–D) DB tumor growth curves in NOG mice from Day 1 to Day 22 after administration of (A) 5 mg/kg ctrl IgG, (B) 2 mg/kg Pola, (C) 3 mg/kg Pola, or (D) 5 mg/kg Pola (*n* = 6). (E–H) #5‐1 tumor growth curves in NOG mice from Day 1 to Day 22 after administration of (E) 5 mg/kg ctrl IgG, (F) 2 mg/kg Pola, (G) 3 mg/kg Pola, or (H) 5 mg/kg Pola (*n* = 6). (I) Tumor regression in scid mice exhibiting DB and #5‐1 tumor regression on Day 22 following Pola administration. Tumor growth curves are those shown in Figures 1A and 4B. (J) Tumor regression in NOG mice exhibiting DB and #5‐1 tumor regression on Day 22 following Pola administration. Tumor growth curves are those shown in Figure 5A–H.

To determine if this lower innate immune cell infiltration explains #5‐1 tumors' reduced Pola sensitivity, we compared the effects of Pola treatment in DB and #5‐1 xenograft models in a NOG mouse model, which lacks mature NK cells and has reduced MΦ function. We found that, whereas 2 mg/kg Pola elicited regression of DB tumors in six out of six mice in the scid mouse model (Figures 1A and 5I), in the NOG mouse model, 2 mg/kg Pola elicited regression of DB tumors in none of the mice (Figure 5B,J). Furthermore, whereas in the scid mouse model, there was a marked difference between the regression of DB tumors and the regression of #5‐1 tumors elicited by 2 mg/kg Pola (Figures 1, 4, and 5I), in the NOG mouse model, there was no difference between the responses of DB and #5‐1 tumors to Pola (Figure 5A–H,J).

These results suggest that the differences between the effect of Pola on DB tumors and its effect on #5‐1 tumors were caused by differences in levels of Pola‐induced immune cell infiltration.

### 3.5 Innate Immune Cells Contribute to the Antitumor Effect of Pola Also in a Syngeneic Mouse Model

**FIGURE 6:**
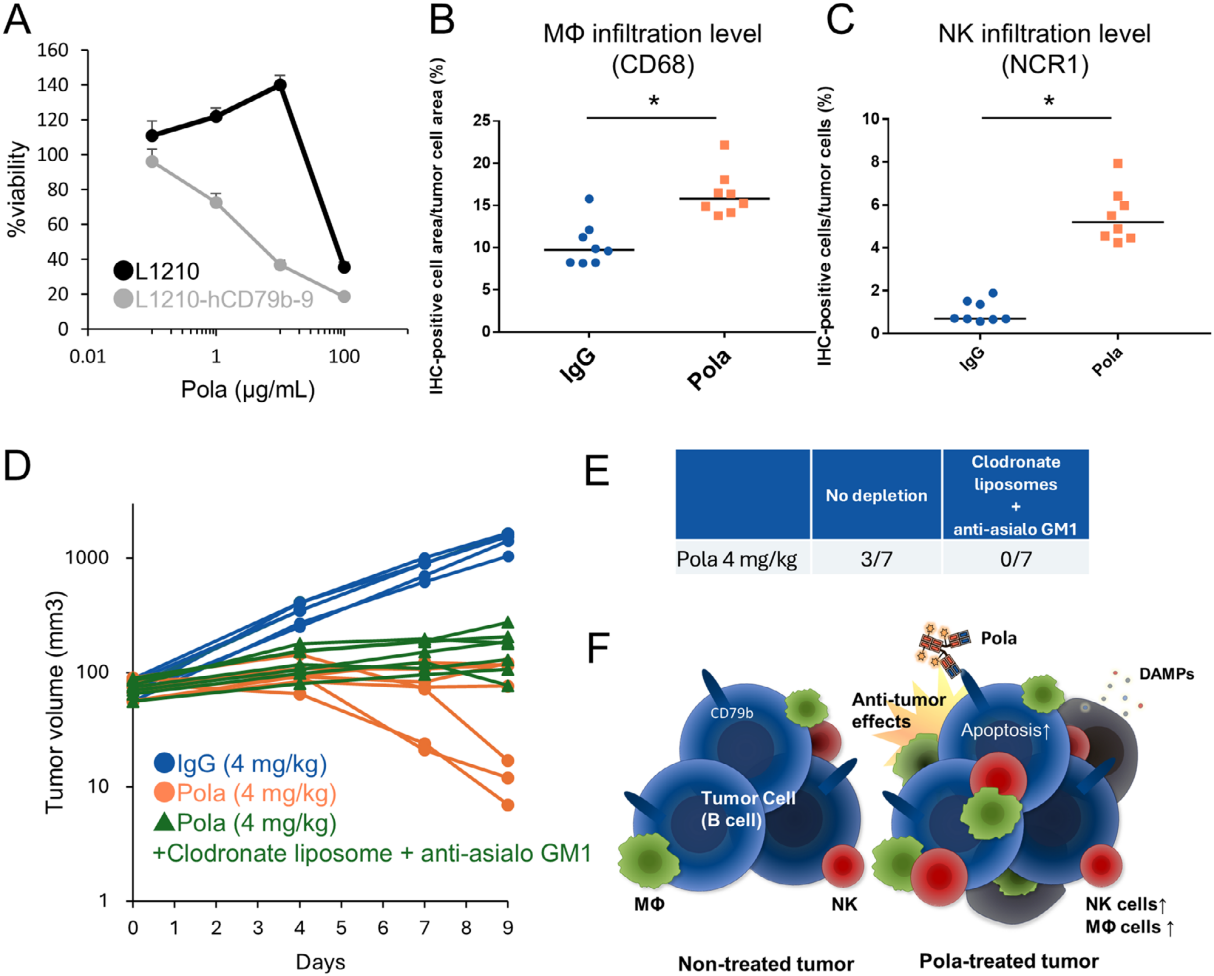
Pola‐induced infiltration of MΦs and NK cells contributes to the antitumor effect of Pola in the L1210‐hCD79b‐9 syngeneic mouse model. (A) Sensitivity of L1210 and L1210‐hCD79b‐9 cells to Pola at the indicated concentrations (3 replicates). The data represent the mean + SD. (B) Percentage of the area of CD68‐positive staining cells to the total area of tumor cells and (C) percentage of NCR1‐positive cells among tumor cells after 4 mg/kg ctrl IgG or 4 mg/kg Pola administration in the L1210‐hCD79b‐9 syngeneic mouse model (*n* = 8). *p < 0.05 by Student's *t*‐test. In dot plots, horizontal bars represent median values. (D) L1210‐hCD79b‐9 tumor growth curves from Day 0 to Day 9 after administration of 4 mg/kg ctrl IgG, 4 mg/kg Pola, or 4 mg/kg Pola plus clodronate liposomes plus anti‐asialo GM1 (*n* = 6–7). (E) Numbers of mice presenting L1210‐hCD79b‐9 tumor regression on Day 9 following Pola administration. In vivo experiments (B–E) were conducted in DBA/2J mice. (F) Schematic diagram illustrating the relationship between the antitumor effect of Pola and innate immune cells.

To study how innate immunity affects Pola efficacy in immunocompetent mice, we established L1210‐hCD79b‐9 cells, which were sensitive to Pola in vitro (Figure 6A).

In the L1210‐hCD79b‐9 syngeneic mouse model, levels of intratumoral infiltration of MΦs and NK cells were higher in the Pola treatment group than in the ctrl IgG group on Day 4 (Figure 6B,C), as was observed in the DB xenograft model. Administration of clodronate liposomes plus anti‐asialo GM1 decreased the number of mice presenting regression of L1210‐hCD79b‐9 tumors elicited by Pola (4 mg/kg) on Day 9 (Figure 6 D,E).

## 4 Discussion

This study analyzed the relationship between the antitumor effect of Pola and the innate immune status in mouse models.

In vivo experiments in the DB xenograft model revealed that Pola increased intratumoral infiltration of MΦs and NK cells (Figures 1, 4, and 4D). In vitro experiments in this study also suggested that Pola‐treated DB cells release DAMPs (Figure 3A–C), similar to findings with other vedotin‐based ADCs [8, 9, 10]. Figure S4 also showed that the cytotoxic effect of Pola's MMAE component appears to be the primary driver of tumor microenvironment changes. A previous study showed that apoptotic cell supernatants attract phagocytes in vivo, with ATP being a key molecule in this recruitment process [22]. In this study, Pola treatment of DB cells enhanced the migration ability of CD45^+^ cells (Figure 3D). Therefore, the infiltration of MΦs and NK cell into tumors could be caused by DAMPs released during Pola‐induced cell death. Our data suggested that Pola‐induced infiltration of immune cells could contribute to the antitumor effect of Pola on DB tumors (Figure 1C,D). Based on the data in Figure 2A,B, the increase in total immune cell numbers, rather than enhanced activation of individual immune cells, appears to be the key driver of anti‐tumor responses. Moreover, the interplay between Pola and innate immune cells also plays a role in the anti‐tumor effect of Pola in the syngeneic model (Figure 6B–E). Unlike conventional chemotherapy that indiscriminately attacks both malignant and immune cells [23], Pola's antibody‐drug conjugate design could enable specific delivery of MMAE to B‐lymphoma cells. This targeted approach potentially minimizes cytotoxic effects on surrounding immune cells within the tumor microenvironment. We cannot definitively determine whether the observed immunomodulatory effects are specific to ADCs or represent a broader class effect of chemotherapeutic agents because this study did not include a non‐ADC chemotherapy control group. Further research should compare the immunomodulatory effects of ADCs like Pola with conventional chemotherapy.

Immune contexture analysis in the POLARIX trial showed that low baseline levels of intratumoral M1 MΦs were associated with poor efficacy of R‐CHOP but not Pola‐R‐CHP [24]. This result might be explained by Pola eliciting high levels of intratumoral immune cell infiltration, thereby maintaining the efficacy of Pola‐R‐CHP, even in patients with low baseline M1 MΦs. Rit, a component of the Pola‐R‐CHP regimen, has an immune‐related mechanism of action targeting B lymphoma cells through processes such as ADCC/ADCP, whereby MΦs or NK cells bind to the antibody‐coated cancer cells [25]. There is a possibility that Pola could enhance the antitumor effect of Rit‐induced ADCC/ADCP because of the increase in the number of innate immune cells elicited by Pola. Moreover, innate immune cells could act cooperatively with acquired immune cells in the tumor microenvironment [26]. Therefore, innate immune cells infiltrated by Pola could have the potential to aid the efficacy of T cell‐related drugs such as T cell‐engaging bispecific antibodies and CAR‐T cells [27, 28, 29, 30]. Further studies are warranted to evaluate the direct or indirect effect of Pola on the acquired immune status and T cell‐related drug efficacy.

To offer the most appropriate treatment from among several therapeutic options for DLBCL patients, it is important to develop a DLBCL classification system able to predict therapeutic responses. The classical cell‐of‐origin (COO) classifications provide only moderate prognostic value and some new classifications of DLBCL have been proposed [31, 32]. In addition, novel microenvironment‐based DLBCL classification suggests that tumor immune context impacts R‐CHOP treatment outcomes as a prognostic factor independent of COO classification [33]. Our study suggests that innate immune cell infiltration is an important factor in the efficacy of Pola. Based on our findings, traditional COO classification may not sufficiently predict responses to Pola‐containing regimens. Therefore, there is a critical need for new classification systems that incorporate immune parameters to guide precision treatment selection, while further research is needed to identify factors causing differences in immune cell infiltration. To establish new prognostic categories suitable for the evolving treatment landscape of DLBCL treatment, additional clinical and preclinical research, including studies with various human cell lines, is needed to analyze the genetic features of tumors and their immune microenvironment before and after therapeutic intervention.

This study demonstrates that Pola treatment alters MΦ and NK cell infiltration in tumors, highlighting these innate immune cells' essential contribution to Pola's antitumor activity. Results indicate Pola can modify the DLBCL microenvironment, suggesting that variations in innate immune cell status within this environment may influence Pola's efficacy.

## Author Contributions

**Mayu Tomita**: conceptualization, data curation, investigation, methodology, writing – original draft, writing – review and editing. **Natsumi Kawasaki**: conceptualization, data curation, investigation, methodology, writing – original draft, writing – review and editing. **Keigo Yorozu**: data curation, investigation, methodology, pathological evaluation, writing – review and editing. **Chie Kato**: data curation, investigation, methodology, pathological evaluation, writing – review and editing. **Sei Shu**: data curation, investigation, methodology, writing – review and editing. **Mitsue Kurasawa**: data curation, investigation, methodology, writing – review and editing. **Bansho Masutani**: data curation, investigation, methodology, writing – review and editing. **Nicolas Sax**: data curation, investigation, methodology, writing – review and editing. **Yoriko Yamashita‐Kashima**: data curation, investigation, methodology, writing – review and editing. **Shigeki Yoshiura**: conceptualization, supervision, methodology, project administration, writing – original draft, writing – review and editing.

## Funding

The authors have nothing to report.

## Ethics Statement

All animal experiments were reviewed and approved by the Institutional Animal Care and Use Committee at Chugai Pharmaceutical Co. Ltd., an institute accredited by AAALAC International.

## Conflicts of Interest

All authors are employees of Chugai Pharmaceutical Co., Ltd.

## Supporting information




**Supporting Figure 1**: The depletion levels of MΦs and NK cells.
**Supporting Figure 2**: DEGs in the MΦs or the NK cells from DB tumors.
**Supporting Figure 3**: Percentage of antibody‐dependent killing with 0.1 or 1 µg/mL Pola in DB cells (3 replicates). Data represent mean ± SD.
**Supporting Figure 4**: MMAE induced the release of DAMPs from DB cells in vitro.
**Supporting Figure 5**: Manual cell annotation in scRNA‐seq analysis.
**Supporting Figure 6**: Manual cell annotation of MΦ subpopulations in scRNA‐seq analysis.

## Data Availability

All data underlying this study are available from the corresponding author upon reasonable request.
