# Supplementary material for: Innate Immune Cell Infiltration Induced by Polatuzumab Vedotin Contributes to the Antitumor Effect in Mouse Models
Source: EJHaem. 2026 Jan 19;7(1):e70219. doi: 10.1002/jha2.70219 (PMC12814615; doi:10.1002/jha2.70219)
Supplement: Supplementary file 1 — Supporting Figure 1: The depletion levels of MΦs and NK cells. Supporting Figure 2: DEGs in the MΦs or the NK cells from DB tumors. Supporting Figure 3: Percentage of antibody‐dependent killing with 0.1 or 1 µg/mL Pola in DB cells (3 replicates). Data represent mean ± SD. Supporting Figure 4: MMAE induced the release of DAMPs from DB cells in vitro. Supporting Figure 5: Manual cell annotation in scRNA‐seq analysis. Supporting Figure 6: Manual cell annotation of MΦ subpopulations in scRNA‐seq analysis. [file JHA2-7-e70219-s001.docx]

# Supplementary Methods

## Establishment of new cell lines

**#5-1 cell line.** DB cells (5 × 10^6^ per mouse) were inoculated subcutaneously into scid mice. After tumor engraftment, eight mice were treated with 2 mg/kg Pola on Days 1, 22, and 43 (total 3 cycles). Regression of DB tumors was observed in all mice, but five mice showed regrowth of tumors by Day 60. On Day 60, the largest tumor among the five mice was passaged *in vivo* to other mice. After tumor re-engraftment, these mice were treated with 2 mg/kg Pola on Day 1, and tumors were dissociated on Day 22 and cultured *in vitro* as #5-1 cells in the DB culture medium with penicillin–streptomycin (Thermo Fisher Scientific).

**L1210-hCD79b-9 cell line.** A plasmid vector containing a human–mouse chimeric CD79b gene sequence including the Pola-recognizing site of human CD79b [indicated in (16) as an hCD79b peptide recognized by SN8: GCCAGATCGGAGGACCGGTACCGGAATCCCAAAGGTAGTGCTTGTTCGCGGATCTGGCAGAGC] and a gene sequence coding for resistance to G418 was purchased from VectorBuilder. Transformation of L1210 cells with this plasmid was conducted by electroporation and cell selection was conducted in a normal culture medium containing 800 µg/mL G418 (Nacalai Tesque). Cells that could grow under G418 were then single-cell cloned, and one of the single-cell cloned cells was established as the L1210-hCD79b-9 cell line.

## Immunohistochemistry (IHC)

Tumor xenograft tissues on Day 4 were collected and fixed with 10% neutral buffered formalin for approximately 24 h and embedded in paraffin according to standard procedures. Subsequently, thin sections were prepared from the paraffin blocks. After deparaffinization of the tissue sections and activation of the antigens, the endogenous peroxidase activity and endogenous non-specific background activity were blocked with 3% hydrogen peroxide at room temperature for 5–15 min. Tissue sections were incubated overnight at 4°C with primary antibodies: anti-mouse/rat CD68 rabbit polyclonal antibody (1:500; cat. no. ab125212; Abcam), anti-mouse NKp46/NCR1 goat polyclonal antibody (1:500; cat. no. AF2225; R&D Systems), anti-mouse/rat NCR1 (EPR23097) rabbit monoclonal antibody (1:391-586; cat. no. ab233558; Abcam), and anti-granzyme B (E5V2L) rabbit monoclonal antibody (1:450; cat. no. 44153; Cell Signaling Technology). This was followed by incubation at room temperature for 15–30 min with their respective polymer-conjugated secondary antibodies: N-Histofine Simple Stain Mouse MAX-PO (R) (undiluted; cat. no. 414341; Nichirei Biosciences), N-Histofine Simple Stain Mouse MAX-PO (G) (undiluted; cat. no. 414351; Nichirei Biosciences), and SignalStain Boost IHC Detection Reagent (HRP, Rabbit) (undiluted; cat. no. 8114; Cell Signaling Technology). Staining was performed at room temperature using a Dako Liquid DAB + Substrate Chromogen System (1:50; cat. no. K3468; Agilent Technologies) for 4-10 min. All sections were counterstained at room temperature with hematoxylin for 1–2 seconds. In addition, TUNEL staining was performed according to the instructions provided with the In Situ Apoptosis Detection Kit (cat. no. MK500; Takara Bio).

CD68 and NCR1 expression was observed under light microscopy (Nikon Eclipse Ni; Nikon Corporation). CD68-positive cells in tumor tissues were calculated from the ratio of the area of CD68-positive staining cells to the total area of tumor cells, and TUNEL- or NCR1-positive cells in tumor tissues were calculated from the ratio of the number of TUNEL- or NCR1-positive staining cells to the total number of tumor cells in the viable region. GzmB-positive cells among NCR1-positive cells were calculated from the ratio of the number of GzmB-positive staining cells to the total number of NCR1-positive staining cells. These analyses were performed using imaging analysis software (Definiens Tissue Studio [version 3.60]; Definiens). For quantification, pathologists first assessed and determined the positive staining criteria. In accordance with software specifications, pathologists selected either area measurement or cell counting based on the morphological characteristics of the cell types being analyzed. The validity of the software analysis results was also confirmed by the pathologists.

## Antibody-dependent killing assay

DB cells were pre-labelled with a CellTrace Violet Cell Proliferation Kit (Thermo Fisher Scientific). CD45^+^ cells were isolated from the DB tumors by using CD45 (TIL) MicroBeads, mouse, and then co-cultured with the labeled DB cells at an effector/target ratio of 40:1 for 16 h in medium containing Pola plus 10 ng/mL murine IL15 (Peprotech). Next, cells were incubated with anti-mouse and human Fcγ receptor antibody (BD Biosciences) and the fixable viability dye FVD780 (Thermo Fisher Scientific), followed by staining with anti-human CD20 antibody (cat. no. 555623; BD Biosciences). Live DB cells (CD20^+^ and CellTrace–stained cells) were counted with CountBright Absolute Counting Beads (Thermo Fisher Scientific) by using a BD LSRFortessa X‐20 cell analyzer (BD Biosciences) and analyzed with FlowJo v10 software. The percentage of antibody-dependent killing was calculated as follows: 100 × [live tumor cell count (ET0) − live tumor cell count (ET40)] / live tumor cell count (ET0), where ET0 indicates an effector/target ratio of 0:1 and ET40 indicates an effector/target ratio of 40:1.

## scRNA-sequencing: library preparation and sequencing

DB and #5-1 tumors in the 2 mg/kg ctrl IgG-treated group and 2 mg/kg Pola-treated group were excised on Day 3, and single-cell suspensions were obtained by digestion with a Tumor Dissociation Kit, human (Miltenyi Biotec). Then, to focus on the tumor-infiltrating immune cells in our analysis, mouse CD45^+^ cells were enriched from the DB or #5-1 tumors by CD45^+^ cell isolation using CD45 (TIL) MicroBeads, mouse. These single-cell samples were fixed with a Chromium Single Cell Fixed RNA Sample Preparation kit (10x Genomics).

Library preparation and scRNA sequencing were conducted by Takara Bio. Single-cell sequencing libraries were generated using Chromium Fixed RNA Kit, Mouse Transcriptome, 4 rxns x 4 BC (10x Genomics) and Chromium X (10x Genomics) according to the manufacturer’s instructions (CG000527_Chromium_FixedRNAProfiling_MultiplexedSamples_UserGuide). The library was sequenced with the NovaSeq 6000 system (Illumina) using paired-end reads.

## scRNA-sequencing: analysis

The mkfastq command in Cellranger v7.1.0 and bcl2fastq2 v2.20 was used to convert the raw fastq records into single-cell RNA sequencing (scRNA-seq) data. All computational analyses on the count matrices were conducted on an Amazon EC2 instance equipped with 48 Intel Xeon Platinum 8259CL CPUs operating at 2.50 GHz, with 186 GB of RAM and an NVIDIA Tesla T4 GPU with 16 GB of memory and support for CUDA 12.

scRNA-seq data preprocessing was performed using Scanpy (version 1.9.8). Initial quality control procedures involved the exclusion of cells expressing fewer than 1% of all detected genes or cells whose mitochondrial genes occupied more than 1% of the total reads, as well as the removal of genes detected in fewer than 0.1% of cells.

Dimensionality reduction was accomplished using scVI (version 1.0.4). The maximum epoch was set to 100, and n_hidden was set to 68. Default values were used for all other parameters. These embeddings were then used to create UMAP (Uniform Manifold Approximation and Projection) plots.

Normalized expression matrices (total count = 10,000) derived from scVI were subjected to Leiden clustering (n_neighbors = 20, resolution = 0.2, min_dist = 0.3) via Scanpy to uncover distinct subpopulations (**Supplementary Fig. S5A**). Marker genes were identified to annotate the cell types of these clusters (**Supplementary Fig. S5B, S5C).** For this purpose, we manually selected marker genes from the PanglaoDB database (https://panglaodb.se/markers.html). Given that PanglaoDB is primarily based on 3′ protocol data and our datasets were generated using the 10x Flex protocol, this manual selection was carried out to choose marker genes that had a reasonable indication of the cell types within these clusters. Macrophage subpopulations were annotated using the same approach (**Supplementary Fig. S6A, S6B**).

Pseudo-bulk expression analysis was conducted by aggregating raw unique molecular identifier (UMI) counts by cell type and experimental batch. Cells with UMI counts more than the 99th percentile were excluded to reduce the influence of potential experimental artifacts, such as cell doublets.

DESeq2 (version 1.42.0) was used to analyze the pseudo-bulk data to identify DEGs in each cell type under different experimental conditions (e.g., comparison between DB and #5-1 strains under IgG treatment). An adjusted *P*-value (with Benjamini–Hochberg correction) threshold of 0.05 with log fold change more/less than 0.25/−0.25 was set to ascertain statistical significance for multiple hypothesis testing.

Overrepresentation analysis was conducted on the DEGs using gseapy (version 1.1.1) for each cell type and each comparison condition. The analysis selected the Gene Ontology terms relevant to this study, such as “cytokine production” and “macrophage activation.” Enriched pathways were determined using an adjusted *P*-value threshold of 0.1, with Benjamini–Hochberg correction, to validate the significance of the enrichment.

# Supplementary Figures


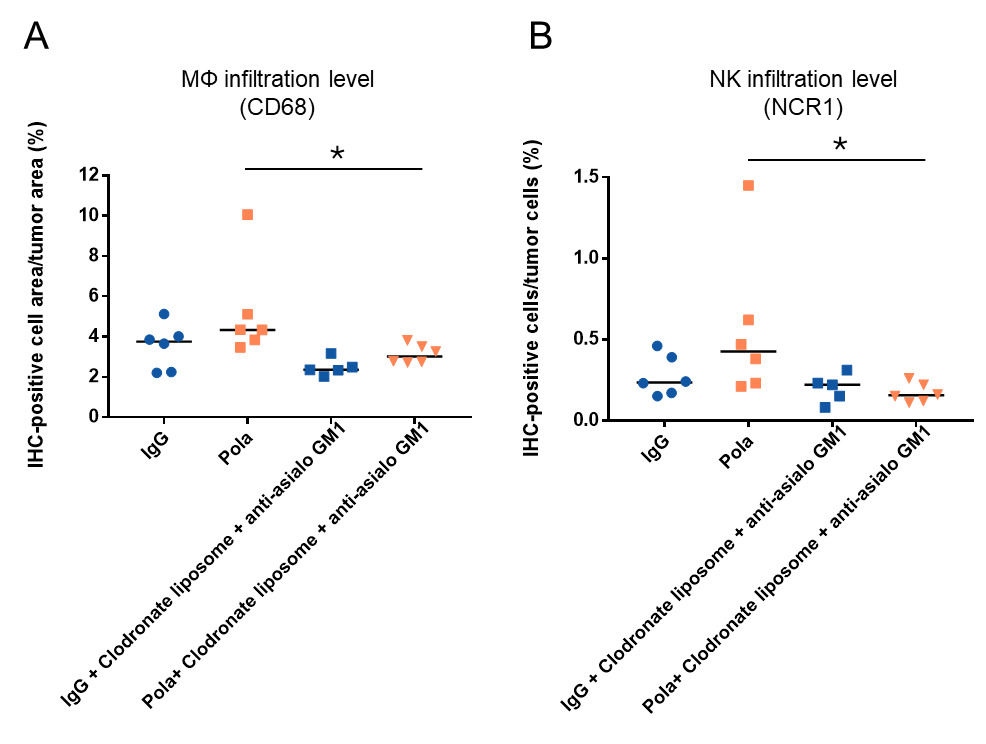


**Fig. S1.** The depletion levels of MΦs and NK cells.

**(A)** Percentage of the area of CD68-positive staining cells to the total area of tumor cells and **(B)** percentage of NCR1-positive cells among tumor cells in the 2 mg/kg ctrl IgG or 2 mg/kg Pola treatment group in mice administered either control liposomes plus normal rabbit serum or clodronate liposomes plus anti-asialo GM1 (*n* = 5–6). * P < 0.05 by Student’s t-test. In dot plots, horizontal bars represent median values. *In vivo* experiments were conducted in scid mice.


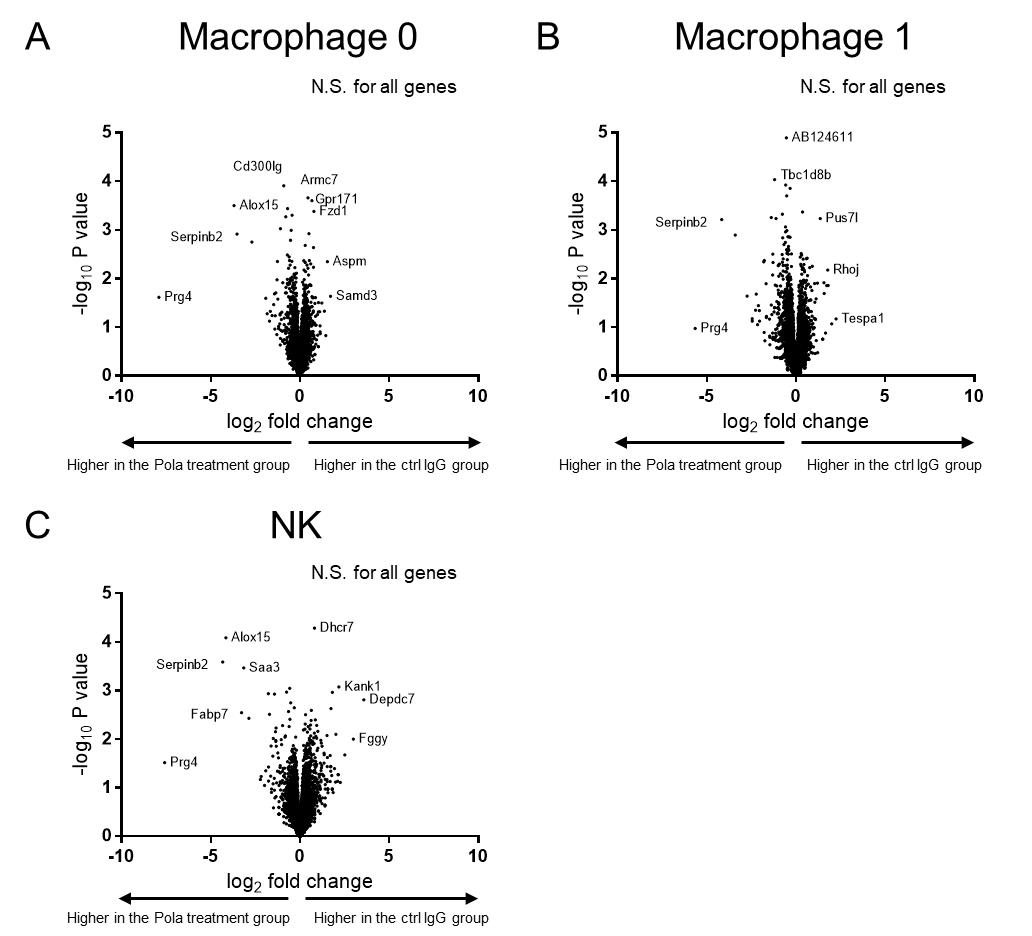


**Fig. S2.** DEGs in the MΦs or the NK cells from DB tumors.

Volcano plots showing the comparison of gene expression between Pola-treated and IgG-treated groups in **(A)** macrophage 0, **(B)** macrophage 1 and **(C)** NK cells from DB tumors. Each dot represents a gene, with the x-axis showing log_2_ fold change and the y-axis showing -log_10_ p-value. A total of 10434 genes were analyzed in the scRNA-seq dataset, with separate analyses performed for each cell type. Since the adjusted p-values are close to one, we used the raw p-values for visualization purposes. An adjusted *P*-value threshold of 0.05 with log fold change more/less than 0.25/−0.25 was set to ascertain statistical significance for multiple hypothesis testing.; N.S.: not significant.

**
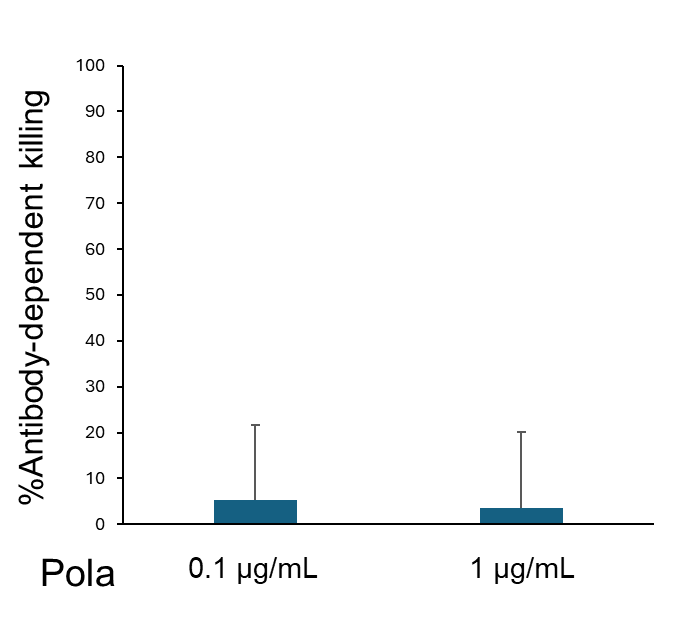
**

**Fig. S3.** Percentage of antibody-dependent killing with 0.1 or 1 µg/mL Pola in DB cells (3 replicates). Data represent mean + SD.

**
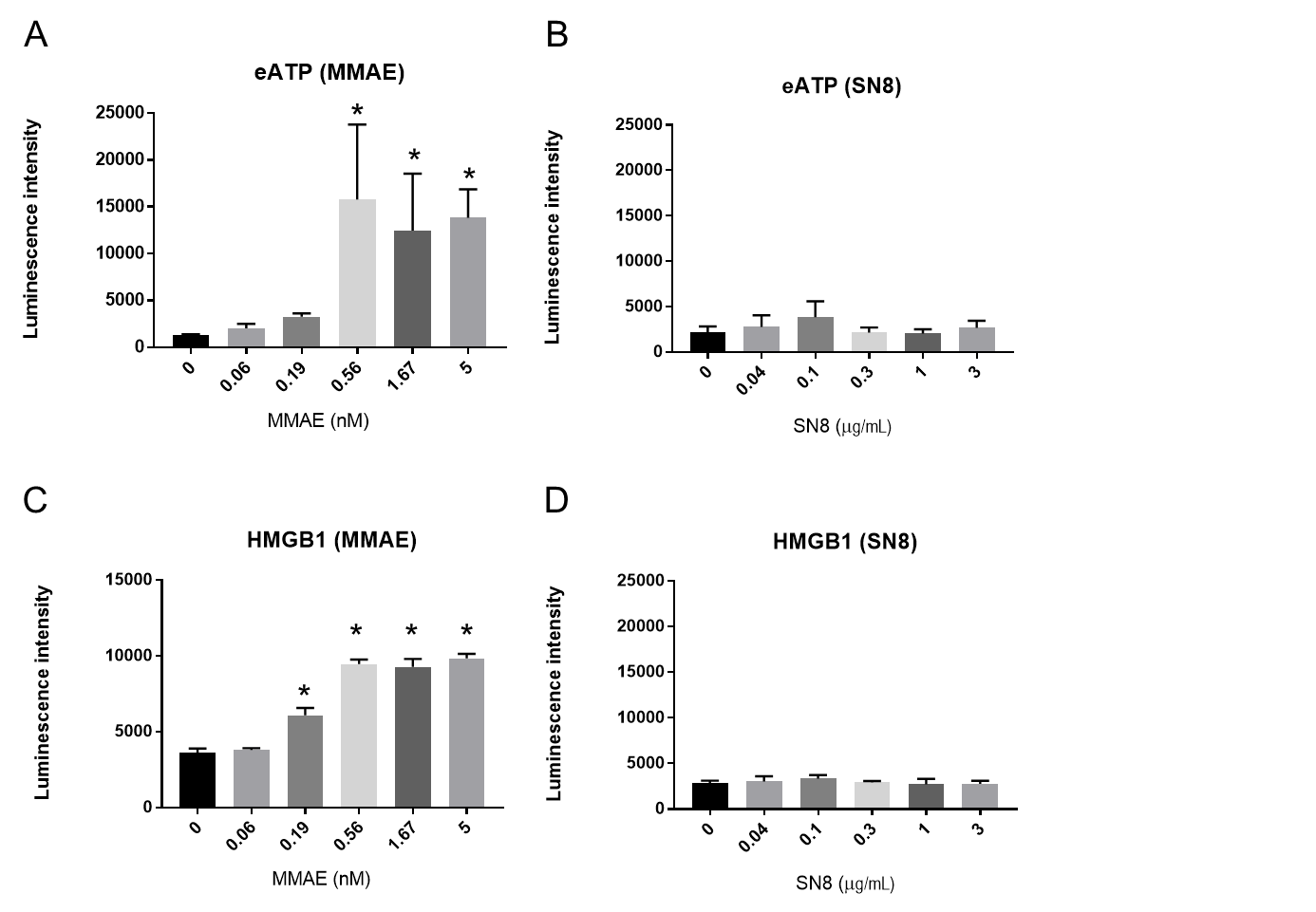
**

**Fig. S4.** MMAE induced the release of DAMPs from DB cells *in vitro*.
Levels of eATP (3 replicates) released from DB cells 2 days after **(A)** MMAE or **(B)** SN8 treatment *in vitro*. Levels of HMGB1 (3 replicates) released from DB cells 3 days after **(C)** MMAE or **(D)** SN8 treatment *in vitro*. The data represent the mean + SD; * *P* < 0.05 by Dunnet’s test compared to MMAE 0 nM or SN8 0 µg/mL.


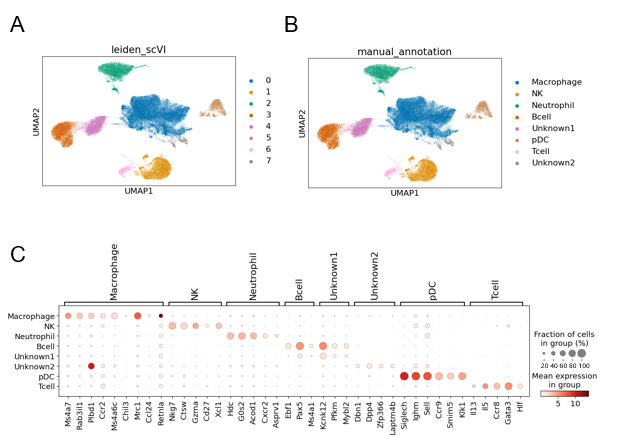


**Fig. S5.** Manual cell annotation in scRNA-seq analysis.
**(A)** UMAP plot of distinct subpopulations by Leiden clustering. **(B)** UMAP plot of the clusters with cell type annotation. **(C)** Marker genes used to annotate the cell types.

**
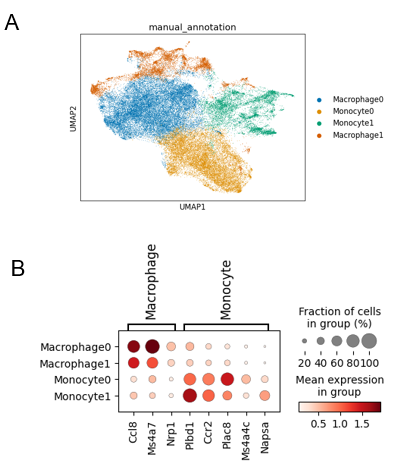
**

**Fig. S6.** Manual cell annotation of MΦ subpopulations in scRNA-seq analysis.
**(A)** UMAP plot of the clusters with subpopulation annotation of MΦs. **(B)** Marker genes used to annotate the MΦ subpopulations.
